# MetaCon: unsupervised clustering of metagenomic contigs with probabilistic k-mers statistics and coverage

**DOI:** 10.1186/s12859-019-2904-4

**Published:** 2019-11-22

**Authors:** Jia Qian, Matteo Comin

**Affiliations:** Department of Information Engineering, University of Padova, Via Giovanni Gradenigo 6, Padova, Italy

**Keywords:** Metagenomics, Unsupervised clustering, K-mers statistics

## Abstract

**Motivation:**

Sequencing technologies allow the sequencing of microbial communities directly from the environment without prior culturing. Because assembly typically produces only genome fragments, also known as contigs, it is crucial to group them into putative species for further taxonomic profiling and down-streaming functional analysis. Taxonomic analysis of microbial communities requires contig clustering, a process referred to as binning, that is still one of the most challenging tasks when analyzing metagenomic data. The major problems are the lack of taxonomically related genomes in existing reference databases, the uneven abundance ratio of species, sequencing errors, and the limitations due to binning contig of different lengths.

**Results:**

In this context we present MetaCon a novel tool for unsupervised metagenomic contig binning based on probabilistic k-mers statistics and coverage. MetaCon uses a signature based on k-mers statistics that accounts for the different probability of appearance of a k-mer in different species, also contigs of different length are clustered in two separate phases. The effectiveness of MetaCon is demonstrated in both simulated and real datasets in comparison with state-of-art binning approaches such as CONCOCT, MaxBin and MetaBAT.

**Electronic supplementary material:**

The online version of this article (10.1186/s12859-019-2904-4) contains supplementary material, which is available to authorized users.

## Introduction

Studies in microbial ecology commonly experience a bottleneck effect due to difficulties in microbial isolation and cultivation [1]. Due to the difficulty in culturing most organisms in a laboratory, alternative methods to analyze microbial diversity are commonly used to study community structure and functionality.

One such method is the sequencing of the collective genomes (metagenomics) of all microorganisms in an environment [2]. Metagenomics is a study of the heterogeneous microbes samples (e.g. soil, water, human microbiome) directly extracted from the natural environment with the primary goal of determining the taxonomical identity of the microorganisms residing in the samples. It is an evolutionary revise, shifting focuses from the individual microbe study to a complex microbial community. As already mentioned in [3, 4], the classical genomic-based approaches require the prior clone and culturing for the further investigation. However, not all bacteria can be cultured. The advent of metagenomics succeeded to bypass this difficulty.

To further investigate the taxonomic structure of microbial samples, assembled sequence fragments, also known as contigs, need be grouped into bin that ultimately represent genomes. Contig binning serves as the key step toward taxonomic profiling and downstream functional analysis. Therefore, accurate binning of the contigs is an essential problem in metagenomic studies.

Grouping contigs into bins of putative species is one of the hurdles faced when analyzing metagenomic data. Typically, one of a few issues are encountered including: struggling to differentiate related microorganisms, repetitive sequence regions within or across genomes, sequencing errors, and strain-level variation within the same species, decreasing accuracy for contigs below a size threshold, or excluding low coverage and low abundance organisms [5, 6].

Despite extensive studies, accurate binning of contigs remains challenging [7]. One category is reference-based (supervised), that is, reference databases are needed for the assignment from contigs or reads to meaningful taxons. The classification is either based on homology, or genomic signatures such as oligonucleotide composition patterns and taxonomic clades. Among the most important methods we can recall: Megan [8], Kraken [9], Clark [10], SKraken [11], and MetaPhlan [12].

Reference-based methods require to index a database of target genomes, e.g. the NCBI/RefSeq databases of bacterial genomes, that is used to classify. These methods are usually very demanding, requiring computing capabilities with large amounts of RAM and disk space. Yet, query sequences originating from the genomes of most microbes in an environmental sample lack taxonomically related sequences in existing reference databases. Most bacteria found in environmental samples are unknown and cannot be cultured and separated in the laboratory [13]. For these reasons, when using reference-based methods the number of unassigned contigs can be very high [14, 15].

The other category of methods is reference-free (unsupervised), where studies extract features from contigs to infer bins based on sequence composition [16–18], abundance [19], or hybrids of both sequence composition and abundance [5, 20–22]. Therefore, these approaches can be applied to bin contigs from incomplete or uncultivated genomes. Some hybrid binning tools, such as CONCOCT [5], MaxBin2.0 [20] and GroopM [21], are designed to bin contigs based on multiple related metagenomic samples. Among these methods, GroopM [21] is advantageous in its visualized and interactive pipeline. On one hand, it is flexible, allowing users to merge and split bins, on the other hand, in the absence of expert intervention, the automatic binning results of GroopM is not as satisfactory as CONCOCT [5]. CONCOCT [5] makes use of the Gaussian mixture model (GMM) to cluster contigs into bins. MetaBAT [22] calculates integrated distance for pairwise contigs and then clusters contigs iteratively by modified K-medoids algorithm. MaxBin [20] compares the distributions of distances between and within the same genomes.

The composition of DNA, in terms of its constituent *k*-mers, is known to be a feature of the genome. All the above studies are based on the assumption that the *k*-mer frequency distributions of long fragments or whole genome sequences are unique to each genome. More precisely, contig binning using k-mers composition is based on the observation that relative sequence compositions are similar across different regions of the same genome, but differ between distinct genomes.

In general, current tools, use the simple *k*-mers counts as signature for a genome, and these counts are normalized, for ease of comparison, in a global fashion. That is all k-mers counts are normalized in the same way, irrespective of the contig/species they belong to. Moreover, when the similarity of two contigs is evaluated as the distance of the corresponding k-mers counts vectors, not all k-mers contributed uniformly to the distance. In fact, because k-mers have different probability to appear, the most probable k-mers can produce a bias in the distance. In this study, we consider this important observation in order to develop a signature based on k-mers statistics that accounts for the different probability of appearance of a k-mer in different species. In general, the pairwise comparison of two sequences, or sets of sequences, can be performed with sophisticated similarity measures, based on k-mers statistics, derived from research in alignment-free statistics [23–28].

Another important aspect is that long contigs carry more information than short contigs. For this reason long contigs, being more representative, they can be effectively grouped into clusters of candidate species, whereas short contigs are often more noisy.

In this paper, we propose MetaCon a method for contig binning based on a new self-standardized k-mers statistics. The main contributions of MetaCon are the following: it learn the different k-mers distributions based on the k-mers probabilities in each contig; it uses the information carried by long contigs to guide the construction of clusters; it can estimate the number of species with a simple iterative procedure. A recent independent benchmark [7] has reported as the best binning methods CONCOCT [5] and MetaBat [22]. We tested MetaCon on simulated and real metagenomes and compared the accuracy of binning with CONCOCT [5], MetaBat [22] and MaxBin [20]. MetaCon performs better in terms of precision, recall and estimated number of species on both simulated and real datasets. The results of this study find that MetaCon can generate high-quality genomes from metagenomics datasets via an automated process, which will enhance our ability to understand complex microbial communities.

## Materials and methods

In this section we present MetaCon and our contribution to the problem of metagenomic contig binning. As we have already observed, most binning tools are based on similarity measures between contigs that are built over k-mers frequency distributions.

However, when dealing with a similarity measure based on k-mers counts there are two major issues. The first is that k-mers might have a different probability to appear in different genomic sequences. The second is that longer contigs carry more information than short contigs, and the direct comparison of the two can be challenging.

**Fig. 1:**
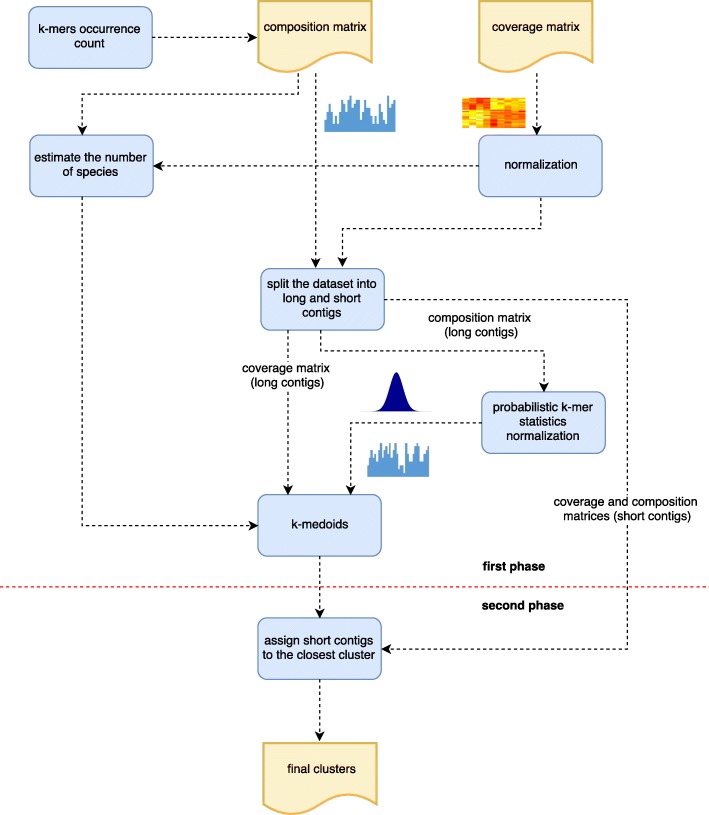
Overview of the MetaCon pipeline

The first problem has been extensively studied in the field of alignment-free measures. The latter, suggest that short contigs should be treated differently. MetaCon addresses these problems by proposing a two-phases binning structure in which each phase process one portion of the input dataset, Fig. 1 shows the processing pipeline of MetaCon. We will describe the main steps of the method, giving a brief explanation of the reasons why they were undertaken. In the following subsections each step will be described in details.

### Contigs representation

Let us assume that we have *N* contigs in input that we need to group into bins. First, we construct the feature matrix, using the same notation as [5], where every row corresponds to a single contig that is represented by a (*M*+*V*) feature vector where *M* is the number of features for the coverage and *V* is the number of features for the composition matrix. This feature matrix has size Nx(M+V), and the two sets of features can be computed independently as follows. Similar to CONCOCT [5], the coverage matrix *Y* represents the average coverage of contigs in every data sample. More precisely, Y is a NxM matrix where *Y*_*cm*_ indicates the coverage of the *c*-th contig in the *m*-th sample. The composition matrix Z of size NxV represents the frequency of every *k*-mers and its reverse complement for the input contigs.

To avoid zero values, a pseudo value is added. For the composition matrix, we add one to it (a relative small number since this matrix counts k-mers frequencies), e.g., $Z_{ij}^{\prime }=Z_{ij}+1$, while for the coverage matrix we modified as $Y_{ij}^{\prime }=Y_{ij}+0.01$ (a negligible quantity in terms of coverage).

In order to normalize the coverage matrix, we re-scale it into different ways. Firstly, across the contigs:

$$\begin{array}{@{}rcl@{}} Y_{cm}^{\prime\prime} = \frac{Y_{cm}^{\prime}}{\sum_{c=1}^{N}Y_{cm}^{\prime}} \end{array} $$

And it is followed by a normalization across samples, within every contig. The coverage profile matrix after this operation is indicated by Q:

$$\begin{array}{@{}rcl@{}} Q_{cm}=\frac{Y_{cm}^{\prime\prime}}{\sum_{m=1}^{M}Y_{cm}{\prime\prime}} \end{array} $$

Each contig *x*_*c*_ is represented by the M coverage features *Q*_*cm*_, with 1≤*m*≤*M* and 1≤*c*≤*N*. These normalizations of the coverage matrix have been already used in CONCOCT [5], and other methods.

In this paper we are interested in building a better feature vector for the k-mer signature, which serves for the following procedure. We observe that the length of contigs plays an important role with respect to the quality of the k-mer signature. Indeed, short contigs may not be a good representer for the genome as they do not carry too much information about the genome, they may not capture the different distributions of k-mers as well as long contigs. Furthermore, since the clustering method (e.g., k-medoids) starts from random contigs as centroids, if it happens to be the short contigs, the clustering performance will somewhat degrade. We try to address this issue by splitting the whole dataset into two parts, based on contig lengths. Long contigs will be clustered in the first phase, whereas short contigs will be treated in the second phase.

### Phase 1: self-standardized k-mers statistics

Inspired by the recent developments in the field of alignment-free statistics we propose here a novel similarity measure based on probabilistic k-mers statistics for the comparison of two contigs. The idea is to account for the different distribution of *k*-mers counts, in different contigs, and to remove the bias generated by contigs of different length in a probabilistic framework with a self-standardized k-mers statistics. Note that this only applies on the long contigs, whereas we do nothing for the short contigs.

Let’s define contig *x*_*c*_, as a sequence of characters from the alphabet *Σ*={*A,C,G,T*}. Let’s say *X*_*cw*_ is the frequency of the *k*-mer *w* in the contig *x*_*c*_. Given that contigs are sequenced from both strands of a genome, *X*_*cw*_ also includes the contribution of the reversed complement of *w*. If *k* is smaller than the logarithm of the length of contigs, *k*<*log*|*x*_*c*_|, we can consider the variables *X*_*cw*_ as Binomial, in line with other studies [29, 30]. Similarly to other methods [22], MetaCon will use *k*=4, as described in result section, thus this approximation holds.

To account for the different probability of appearance of *k*-mers, we standardize the variables *X*_*cw*_ in the following way. For the sequence *x*_*c*_, let $p_{c}^{j}(a)$ be the probability of the symbol *a* in position *j* in *x*_*c*_. If we assume that the symbols at different positions are independent and identically distributed, we can simplify $p_{c}^{j}$ and denote it by *p*_*c*_. This i.i.d. model has been widely used in field of pattern statistics [31, 32]. Based on this assumption, we define the probability of a *k*-mer *w*=*w*_1_*w*_2_...*w*_*k*_ to occur at a given position in the contig *x*_*c*_ as $P_{cw}=\prod _{i=1}^{k}{p_{c}(w_{i})}$, that again is independent of the position of *x*_*c*_.

Now, we recall that *X*_*cw*_ is a Binomial and that the k-mer *w* has probability to occur *P*_*cw*_, thus can compute mean and variance of *X*_*cw*_ as:

1 $$\begin{array}{@{}rcl@{}} E[X_{cw}] = \mu_{cw} = P_{cw} L(x_{c}) \end{array} $$

2 $$\begin{array}{@{}rcl@{}} Var(X_{cw}) = (\sigma_{cw})^{2} = P_{cw}(1 - P_{cw}) L(x_{c}) \end{array} $$

where *L*(*x*_*c*_) is the length of the contig *x*_*c*_. Thus, the k-mers counts *X*_*cw*_ can be standardized, as a z-score, as follows:

3 $$\begin{array}{@{}rcl@{}} \widetilde{X}_{cw} = \frac{{X_{cw}} - \mu_{cw}}{\sigma_{cw}} \end{array} $$

As already observed the frequency of *k*-mers in different genomes can greatly vary. Similarly, it is difficult to estimate the probability *P*_*cw*_, as it does not follow the same model for different genomes. Thus we need to estimate *P*_*cw*_ directly from the contig. We define *n*_*c*_(*a*), with *a*∈{*G,T,A,C*}, as the number of occurrences of the nucleotide *a* in the contig *x*_*c*_. Then, we can estimate the probability of the symbol *a* in the contig *x*_*c*_ as,

$$\begin{array}{@{}rcl@{}} p_{c}(a)=\frac{n_{c}(a)} {L(x_{c})} \end{array} $$

To summarize, we start from the raw k-mers counts directly obtained from matrix *Z*^′^, for each contig we can compute the probabilities *P*_*cw*_ and build a probabilistic k-mers statistics $\widetilde {X}_{cw}$ by using formula (3). Similar to the normalization applied to the coverage features, the probabilistic k-mers statistics $\widetilde {X}_{cw}$ is column-wise normalized (normalization across contigs), as H:

$$\begin{array}{@{}rcl@{}} H_{cw} = \frac{\widetilde{X}_{cw}}{\sum_{c} {\widetilde{X}_{cw}} } \end{array} $$

Finally, the feature matrix F of long contigs is assembled as *F*=[*QH*], as the combination of the coverage profile *Q* and probabilistic k-mers profile H. Then, the relatedness of a pair of contigs can be evaluated by L2 distance of the corresponding feature vectors. Here we use k-medoids clustering method ([33]), a variant of k-means with the feature matrix F as input.

### Phase 2: dealing with short contigs

In the first phase we filter short contigs, and we build clusters with the k-medoids algorithm by using the feature matrix F mentioned above. We process only long contigs in the first phase, because they are more informative in terms of k-mers statistics. We believe that the underlying structure of every species can be well unveiled in the first stage when we get rid of the short contigs from the dataset. In fact, the clusters produced in the first phase will have high precision (see result section), because they are more distinguishable and less noisy. These clusters will be used as a basis for the assignment of short contigs in the second phase.

The second subset contains the short contigs, and we decided to assign them to the already classified clusters (output after the first phase) according to the shortest L1 distance. The profile matrix is the union of the composition and coverage matrices of the short contigs. Note that the composition matrix for short contigs is not normalized. L1 distance is an alternative method to measure the similarities between two multi-dimensional data by computing the absolute distance. In our case, we observed that L1 works better than L2 (Euclidean distance) in the second stage. We think that the L1 distance may somewhat amplify the differences better than L2 in the second-phase where short contigs are less representative.

An overview of MetaCon is presented in Fig. 1. Here we summarize the overall procedure.

1. Compute the composition and coverage matrices.
2. Normalize the coverage matrix.
3. Estimate the number of clusters: C.
4. Split the dataset into two subsets: long and short contigs.
5. First-phase: Compute the probabilistic k-mers signature and normalize the composition matrix of long contigs.
6. Clustering long contigs by k-medoids
  - Initialization: randomly select C contigs as the medoids.
  - Assignment step: Associate each contig to the closest medoid.
  - Update step: For each medoid *m* and each contig *c* associated to *m* swap *m* and *c* and compute the total cost of the new configuration, based on the average dissimilarity of *c* to all contigs associated to *m*. Select the medoid *c* with the lowest configuration cost.
  - Repeat steps b and c until there is no change in the assignments.
7. Second-phase: Assign the short contigs to the closest centroid by L1 distance.

### Estimating the number of species

As we know, estimating the real number of clusters is one of the most challenging problem. The difficulty primarily attributes to the absence of prior knowledge of the data, in the case of metagenomics the real number of species in the dataset is not known. Moreover, there is no general criteria that may well assess the clusters when we encounter different datasets, in particular, when the number of clusters is big and the data has high-dimensional. Despite some methods that are tailored for the datasets with known distribution, here instead we use an easy and intuitive method to estimate the number of species. We exhaustively iterate the k-means by starting from a small number of clusters and gradually increase it until some criteria is met. This procedure stops when the non-empty clusters are less than 80% of the candidate number of cluster in the corresponding iteration. This iterative procedure might be computationally demanding, to speed up the computation in this paper we use an efficient library implementation [34].

## Results and discussion

In order to validate our contribution, we compare it with the commonly known methods CONCOCT, MaxBin 2.0 and MetaBat. In particular, CONCOCT [5] and MetaBat [22] have been reported to be the best performing methods in a recent independent benchmark [7]. All of these tools use as input the composition and coverage matrices, as MetaCon does. MaxBin 2.0 [20] estimates the probability that a contig belongs to a bin based on expectation-maximization (EM). MetaBat [22] starts from one bin, and gradually assigns the contigs to that bin until the centroid does not change, repeatedly for several bins until no contigs are left. CONCOCT [5] applies PCA (principal component analysis) to the feature matrix (composed by coverage and composition matrices) for the sake of dimension reduction and afterward it uses a Gaussian mixture model.

### Synthetic and real datasets

Before the discussion of the results, here we give a brief introduction of the datasets. In this paper, we test the methods on both synthetic and real metagenomic datasets. A complete description of the dataset construction can be found in [5], here for completeness we report a brief summary. In CONCOCT [5], the authors simulate two mocked communities of microbiomes in order to test the performance, called ’Strain’ and ’Species’ datasets. Both of these synthetic datasets are built on 16S rRNA samples involved in the Human Microbiome Project (HMP, [35]). The samples have gone through denoise operation and the OTUs were generated by the standard that 3% sequence differences to approximated species exist. The contigs were assembled from the reads in samples and the reads were subsequently mapped back onto contigs to get the coverage information.

For simulated and real data, a co-assembly of reads from all samples was performed using Ray [36]. Ray was used to generate the co-assembled contigs because it is able to handle large metagenomic dataset. Contigs were cut up into non-overlapping fragments of 10 kilobases in order mitigate the effect of local assembly errors (Additional file 1: Figure S1 in supplementary material reports the contig length distribution).

Specifically, the ’Strain’ dataset contains 9417 contigs, which are co-assembled from 64 samples, and it contains totally 20 organisms. The simulated ’Strain’ community is composed by five different *Escherichia coli* strains, five different *Bacteroides* species, five different *gut* bacteria and the rest from *Clostridium*.

The ’Species’ dataset has 101 different species, including 37628 contigs, co-assembled from 96 samples. For the ’Species’ dataset, OTUs are removed when its total count is less then 20 across samples. This dataset aims at testing the ability to discriminate at species-level. The complete information for the datasets can be found in the supplementary material.

’Sharon’ [37] is a real dataset, and it is generated from the microbiome samples of the premature infants. It contains 18 data samples, and due to the fact that we do not know the true species labels, we used TAXAassign [38] to annotate the contigs. It ended up with 7 species, 2599 contigs, after we filtered the contigs with ambiguous labels at species level.

### Evaluation criteria

Precision and recall are commonly used to compare the performance of the binning algorithms under assessment. Precision measures the ability of the approach to build clusters composed by contigs from the same species. On the other hand, recall measures the ability of gathering all the contigs of a given species. Namely, the precision tests the correctness, and the recall tests the completeness. Therefore, when evaluating the performance of a binning method one should take into account both aspects in order to obtain a comprehensive evaluation.

Let *n* be the number of species in a metagenomic dataset, and *C* be the number of clusters returned by the algorithm. Let *A*_*ij*_ be the number of contigs from species *j* assigned to cluster *i*. Following the definitions in [39], for the precision we find the species with the maximum number of contigs in every cluster and sum them up, divided by the total number of contigs. As for the recall, we select the cluster with the maximal number of contigs from a given species, and again accumulate them, divided by the total number of contigs.

$$\begin{array}{@{}rcl@{}} Precision=\frac{\sum_{i=1}^{C} {max}_{j} A_{ij}}{\sum_{i=1}^{C}\sum_{j=1}^{n} A_{ij}} \end{array} $$

$$\begin{array}{@{}rcl@{}} Recall=\frac{\sum_{j=1}^{n} {max}_{i}A_{ij}}{\sum_{i=1}^{C}\sum_{j=1}^{n} A_{ij}} \end{array} $$

### Results on Synthetic and Real Datasets

In the first experiment, we assess the ability of MetaCon to predict the number of clusters. The average result is reported in (Table 1). CONCOCT needs a maximal number of cluster in input, the other methods do not. In this first experiment, MetaCon outperforms the other methods by estimating the number of clusters close to the real number of species.

**Table 1:** Estimated number of clusters for different methods (best results are in bold)

Dataset	Real value	CONCOCT	MaxBin	MetaCon
Strain dataset	20	**21**	17	**21**
Species dataset	101	84	114	**106**
Sharon dataset	7	**8**	5	**6**

**Fig. 2:**
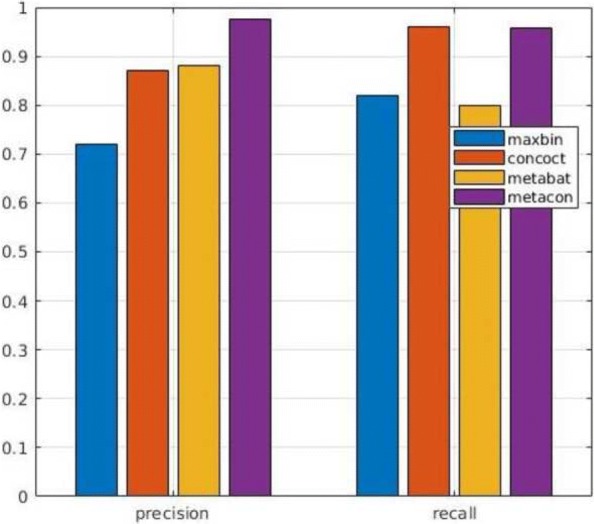
Comparison of precision and recall for **Strain** dataset

**Fig. 3:**
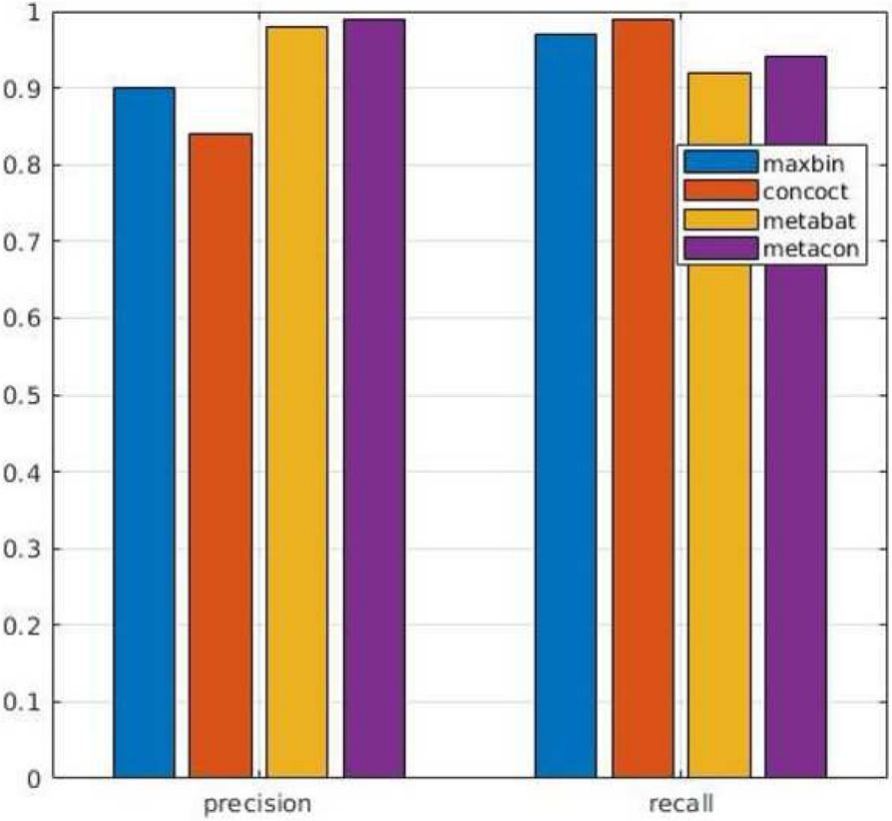
Comparison of precision and recall for **Species** dataset

**Fig. 4:**
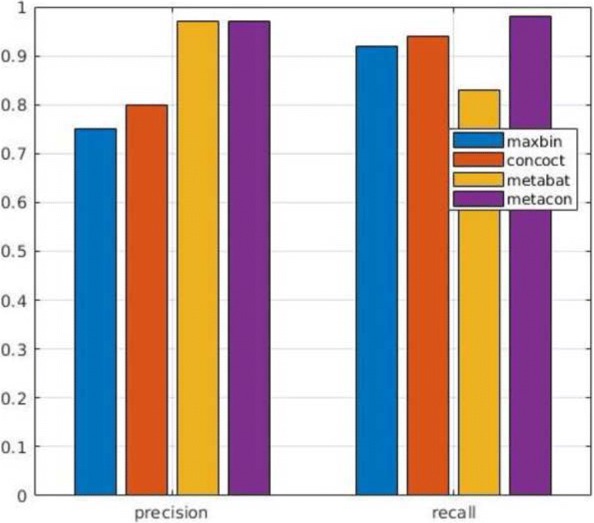
Comparison of precision and recall for **Sharon** dataset

In next series of tests we evaluated the performance of MetaCon on the datasets against the other tools. MetaCon outperforms all other methods in terms of precision and recall, as shown in Figs. 2, 3 and 4. The precision and recall are above 95% for both simulated data and real data. For the ’Strain’ dataset (Fig. 2), the precision by MetaCon is about 97.5*%*, that is better than the other three methods; the recall is 95.8*%*, higher than MaxBin and MetaBat, almost identical with CONCOCT. For the ’Species’ dataset, shown in Fig. 3, it is challenging to bin the contigs since the number of species is large, MetaCon reaches 99.3*%* in terms of precision and 94.6*%* for the recall. Again, the comparison with other tools reveals an outcome similar to the dataset ’Strain’. For the real dataset ’Sharon’, Fig. 4, the results are in line with those of the synthetic datasets. MetaCon achieves higher precision and recall with respect to the other tools. The only notable difference is that on this datasets MetaBat has a precision similar to MetaCon but again a lower recall.

**Fig. 5:**
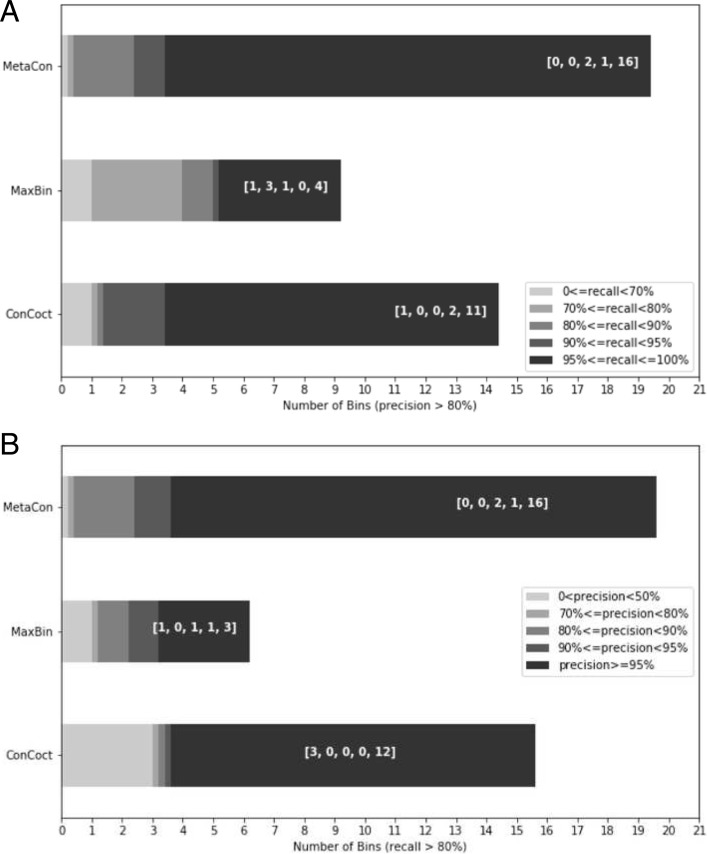
The quality of bins generated by different methods. **a** After filtering out the bins, whose precision is lower than 80%, we compare the recall located in different range for different methods. The array marked in white indicates the number of bins in the corresponding recall. The thin stripes represent absence of bins. **b** After filtering out the bins, whose recall is lower than 80%, we compare the precision located in different range for different methods. The array marked in white indicates the number of bins in the corresponding precision. The thin stripes represent absence of bins

Additionally, we evaluate the quality of bins generated by different methods for Strain dataset. In order to evaluate the contamination and completeness of the bins, we filtered out the bins whose precision is less than 80%, reported in Fig. 5a, where the different shades of gray indicates the different level of recall. In Fig. 5b, we report the opposite procedure where we assess the precision of bins after filtering out bins with recall lower than 80%. For example, in Fig. 5a the number of clusters with precision greater than 80% and recall greater than 95% is 16 for MetaCon, for CONCOCT 11 and for MaxBin 4. MetaCon outperforms the other methods, firstly MetaCon has more bins left after screening in both Fig. 5a and b. Secondly, the bins produced by MetaCon mostly resides in the high-level range of precision and recall. We think that the primary reason for the good performance of MetaCon is that the first-stage builds high-quality clusters, they may better represent the relative species and capture the different traits of species. In addition, the k-medoids may relieve the negative influence caused by the outliers since it considers the median value instead of the mean during the clustering process, and probably it further consolidates the structures of clusters.

### Parameters: k-mers size

In this section, we want to discuss how to choose the parameters *k* for MetaCon and show the results under different conditions. The selection of the k-mers size is critical when we build our probabilistic k-mers signature, if k is too small (k=2), it will result a less representative and informative feature matrix as only 16 features of composition matrix generated, that is not sufficient to differentiate between contigs from diverse species, specially, when some of them are closely related.

**Fig. 6:**
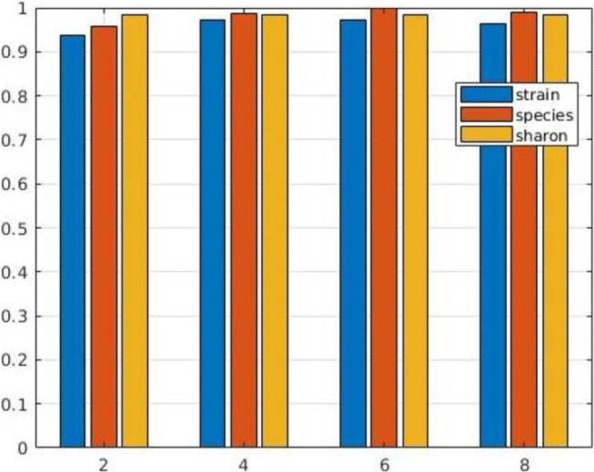
MetaCon precision for different datasets by varying k-mers size

**Fig. 7:**
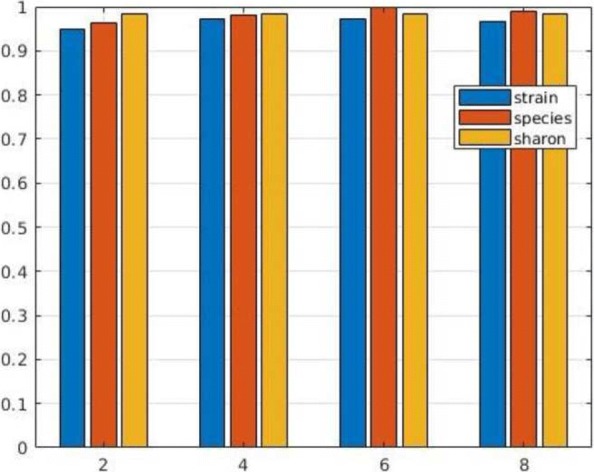
MetaCon recall for different datasets by varying k-mers size

With this series of experiments we want to evaluate the influence of k-mers size for MetaCon over the different datasets. The results of precision and recall are reported in Figs. 6 and 7. Note that the results are obtained with the correct number of clusters used as input since here we want to compare the various choice for the size of k-mers. For the ’Strain’ dataset, the precision increases from 93% to 97% when k varies from 2 to 6, and the precision is identical when k equal to 4 and 6, when k equal to 8 the precision decreases. The recall of ’Strain’ dataset follows the similar trend of the precision. As for the ’Species’ dataset, the precision changes from 95% to 99% when k increases from 2 to 6, and it achieves 99% when k equal to 6, slightly better than with k equal to 4. The ’Sharon’ dataset keeps the precision and recall constant when we modify k as the number of species is small, probably less information is required in order to cluster the contigs with respect to the other two datasets that contain more species. Generally speaking, k equal to 4 could be a good choice, considering precision, recall and computing time. A similar value was used also in MetaBat [22].

### The importance of contig length distribution

Another factor we want to address here is the importance of the length of contigs. Recall that in the first phase of MetaCon, we process only long contigs, and in the second phase we assign the short contigs. We want to evaluate the impact of this approach by showing how precision and recall varies in the two phases and to compare the results when we use all the contigs at once. The results of these experiments are reported in Tables 2 and 3. If we do not process the long and short contigs separately in two-phases we can observed that the precision obtained by MetaCon is respectively 93.79*%* and 97.23*%* for the two datasets ’Strain’ and ’Species’. We can notice that the precision can be improved by separately processing the contigs: for the ’Strain’ dataset, it increases from 93.79*%* to 97.46*%*, and for ’Species’ it improves from 97.23*%* to 99.56*%*. A similar behavior is observed for the recall. For the ’Strain’ dataset the recall increases slightly from 95.23*%* to 95.78*%*, and for the ’Species’ dataset it improves from 90.95*%* to 95.04*%*.

**Table 2:** Precision of MetaCon after the different phases, compared with the precision considering all contigs at once

Dataset	First-phase	Second-phase	All contigs
Strain	98.70%	97.46%	93.79%
Species	99.88%	99.56%	97.23%

**Table 3:** Recall of MetaCon after the different phases, compared with the recall considering all contigs at once

Dataset	First-phase	Second-phase	All contigs
Strain	75.05%	95.78%	95.23%
Species	80.86%	95.04%	90.95%

In order to choose a good threshold to split short and long contigs, we experimented with different values (see Additional file 1: Figure S2 in supplementary material). Empirically we found that a good choice is to have about 20% of the contigs to be labelled as short. Based on these results we selected 2000bp as a good compromise.

### Assignment of short contig: L1 vs L2 distance

**Fig. 8:**
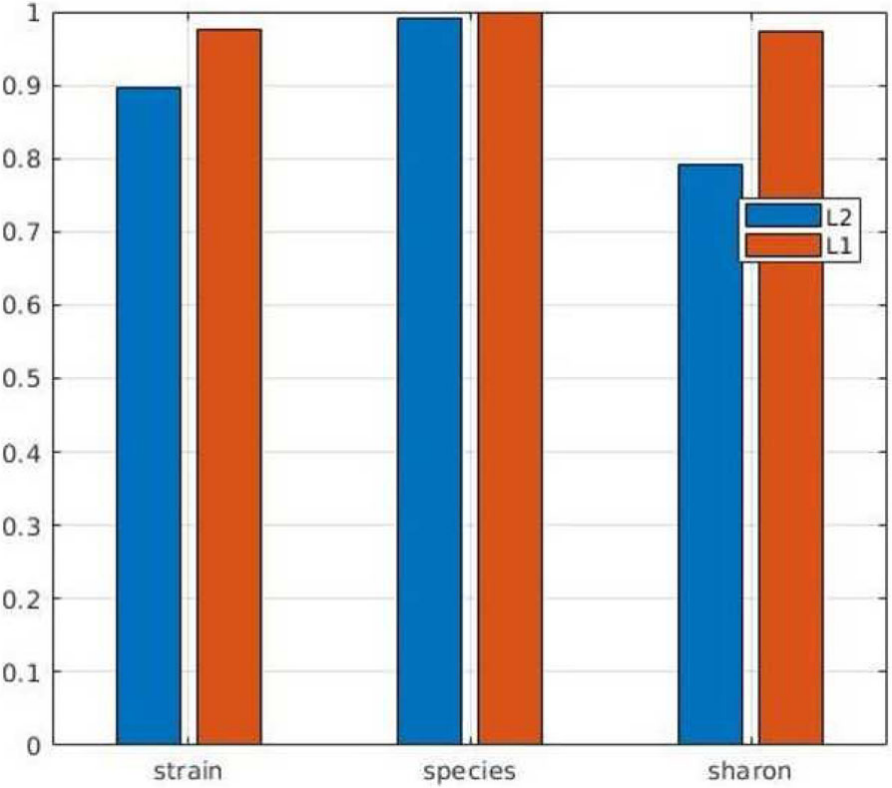
Comparison of L1 and L2 distance in the second-phase of MetaCon

In the second phase short contigs are assigned to the closest centroid by L1 distance. Here, we evaluate the effect of L1 distance in comparison with L2 distance. Figure 8 reports the precision of MetaCon for all datasets individually by L1 and L2 distance in the second phase. For this stage, the L1 distance outperforms L2 for all of the datasets. In particular, in the ’Strain’ dataset L1 boosts the performance from 89.56*%* to 97.46*%*, for ’Sharon’ dataset, the precision increases from 79.11*%* to 97.38*%* by using L1. We think, when it comes to assign the short contigs to the closest cluster centroid, L1 reveals its strength by amplifying the differences between contigs.

## Conclusion

Binning metagenomic contigs remains a crucial step in metagenomic analysis. In this work we presented MetaCon, an unsupervised approach for metagenomic binning based on probabilistic k-mers statistics and coverage. Our approach instead of processing the whole dataset at once as most methods, it splits the input and process them into two separate phases of MetaCon. We compared the binning performance over synthetic and real metagenomic datasets against other state-of-art binning algorithms, showing that MetaCon achieves good performances in terms of precision, recall and estimating the number of species.

## Additional file

Additional file 1 Supplementary Material. (PDF 205 kb)

## Abbreviations

GMM: Gaussian mixture model
HMP: Human microbiome project
OTU: Operational taxonomic unit
PCA: Principal component analysis
