# Supplementary material for: MetaCon: unsupervised clustering of metagenomic contigs with probabilistic k-mers statistics and coverage
Source: BMC Bioinformatics. 2019 Nov 22;20(Suppl 9):367. doi: 10.1186/s12859-019-2904-4 (PMC6873667; doi:10.1186/s12859-019-2904-4)

Supplementary Materials

Table1

Identifier	Species	NCBI Acc_No
ESC_COL_K12	Escherichia coli K-12	NC_000913.3
ESC_COL_O104	Escherichia coli STEC outbreak	NC_018658.1, NC_018659.1, NC_018660.1, NC_018666.1
ESC_COL_O157	Escherichia coli Sakai	NC_002127.1,NC_002128.1,NC_002695.1
ESC_COL_UT189	Escheria coli UPEC	NC_007941.1,NC_007946.1
ESC_COL_O127	Escheria coli ETEC	NC_011601.1,NC_0116026.1,NC_603.1
BAC_VUL	Bacteroides vulgatus ATCC 8482	NC_009614.1
BAC_THE	Bacteroides thetaiotaomicron VPI-5482	NC_004703.1
BAC_FRA	Bacteroides fragilis 638R	NC_016776.1
BAC_HEL	Bacteroides helcogenes P 36-108	NC_014933.1
BAC_SAL	Bacteroides salanitronis DSM 18170	NC_015166.1
CLO_DIF	Clostridium difficile BI1	NC_017179.1
FAE_PRA	Faecalibacterium prausnitzii L2-6	NC_021042.1
ROS_HOM	Roseburia hominis A2-183	NC_015977.1
RUM_ALB	Ruminococcus albus 7	NC_014833.1
EUB_REC	Eubacterium rectale ATCC 33656	NC_012781.1
BIF_LON	Bifidobacterium longum subsp. longum BBMN68	NC_014656.1
ENT_FAE	Enterococcus faecalis D32	NC_018223.1
AKK_MUC	Akkermansia muciniphila ATCC BAA-835	NC_010655.1
LAC_BRE	Lactobacillus brevis ATCC 367	NC_008499.1
HEL_PYL	Helicobacter pylori Rif 1	NC_018937.1

Table2

Identifier	Species	NCBI Acc_No
ACH_LAI	Acholeplasma laidlawii PG-8A	NC_010163
ACI_FER	Acidaminococcus fermentans DSM 20731	NC_013740
ACI_INT	Acidaminococcus intestini RyC MR95	NC_016077
AKK_MAN	Akkermansia muciniphila ATCC BAA 835	NC_0010655
ALI_FIN	Alistipes finegoldii DSM 17242	NC_018011
ALI_SHA	Alistipes shahii WAL 8301	NC_021030
AST_EXC	Asticcacaulis excentricus CB 48	NC_014816,NC_014817,NC_014818,NC_014819
BAI_CER	Bacillus cereus E33L	NC_006274,NC_007103,NC_007104,NC_007105,NC_007106,NC_007107
BAI_THU	Bacillus thuringiensis serovar konkukian 97 27	NC_005957,NC_006578
BAC_FRA	Bacteroides fragilis YCH46	NC_006297,NC_006347
BAC_HEL	Bacteroides helcogenes P 36 108	NC_014933
BAC_THE	Bacteroides thetaiotaomicron VPI 5482	NC_004663,NC_004703
BAC_VUL	Bacteroides vulgatus ATCC 8482	NC_009614
BIF_ADO	Bifidobacterium adolescentis ATCC 15703	NC_008618
BIF_LON	Bifidobacterium longum NCC2705	NC_004307,NC_004943
BRI_BRE	Brevibacillus brevis NBRC 100599	NC_012491
BRE_SUB	Brevundimonas subvibrioides ATCC 15264	NC_014375
BUT_BAC	Butyrate producing bacterium SSC 2	NC_021016
CAN_ACC	Candidatus Accumulibacter phosphatis clade IIA UW 1	NC_013190,NC_013191,NC_013193,NC_013194
CAN_LIB	Candidatus Liberibacter solanacearum CLso ZC1	NC_014774
CAN_PHY	Candidatus Phytoplasma mali	NC_011047
CLA_MIC	Clavibacter michiganensis sepedonicus	NC_010399,NC_010407,NC_010408
CLO_ACI	Clostridium acidurici 9a	NC_018657,NC_018664
CLO_BEI	Clostridium beijerinckii NCIMB 8052	NC_009617
CLO_BOT	Clostridium botulinum H04402 065	NC_017299
CLO_CEL	Clostridium cellulolyticum H10	NC_011898
CLO_CEV	Clostridium cellulovorans 743B	NC_014393
CLO_SAC	Clostridium cf saccharolyticum K10	NC_021047
CLO_CLA	Clostridium clariflavum DSM 19732	NC_016627
CLO_DIF	Clostridium difficile R20291	NC_013316
CLO_PER	Clostridium perfringens SM101	NC_008262,NC_008263,NC_008264,NC_008265
CLO_PHY	Clostridium phytofermentans ISDg	NC_010001
CLO_SAW	Clostridium saccharolyticum WM1	NC_014376
CLO_SPS	Clostridium SY8519	NC_015737
CLO_STE	Clostridium stercorarium DSM 8532	NC_020887
CLO_STI	Clostridium sticklandii	NC_014614
CLO_THE	Clostridium thermocellum DSM 1313	NC_017304
CLO_CAT	Coprococcus catus GD 7	NC_ 021009
COP_ART	Coprococcus ART55 1	NC_021018
COR_GLO	Coriobacterium glomerans PW2	NC_015389
CUP_MET	Cupriavidus metallidurans CH34	NC_007971,NC_007972,NC_007973,NC_007974

CYL_STA	Cylindrospermum stagnale PCC 7417	NC_019744,NC_019757,NC_019758,NC_020050
DEH_SPC	Dehalobacter CF	NC_018867
DES_DIC	Desulfitobacterium dichloroeliminans LMG P 21439	NC_019903
DES_ACE	Desulfotomaculum acetoxidans DSM 771	NC_013216
ECH_VIE	Echinicola vietnamensis DSM 17526	NC_019904
ENT_HIR	Enterococcus hirae ATCC 9790	NC_015845,NC_018081
ERY_RHU	Erysipelothrix rhusiopathiae SY1027	NC_021354
ETH_SHA	Ethanoligenens harbinense YUAN 3	NC_014828
EUB CYL	Eubacterium cylindroides T2 87	NC_021019
EUB_ELI	Eubacterium eligens ATCC 27750	NC_012778,NC_012780,NC_012782
EUB_REC	Eubacterium rectale ATCC 33656	NC_012781
EUB_SIR	Eubacterium siraeum V10Sc8a	NC_021043
FAE_PRA	Faecalibacterium prausnitzii	NC_021020
FIL_ALO	Filifactor alocis ATCC 35896	NC_016630
FUS_NUC	Fusobacterium nucleatum ATCC 25586	NC_003454
HAE_PAR	Haemophilus parainfluenzae T3T1	NC_015964
HER_SER	Herbaspirillum seropedicae SmR1	NC_014323
JAN_MAR	Janthinobacterium Marseille	NC_009659
LAW_INT	Lawsonia intracellularis PHE MN1 00	NC_008011,NC_008012,NC_008013,NC_008014
MEG_ELS	Megasphaera elsdenii DSM 20460	NC_015873
MES_FLO	Mesoplasma florum L1	NC_006055
MOO_THE	Moorella thermoacetica ATCC 39073	NC_007644
MYC_MYC	Mycoplasma mycoides capri LC 95010	NC_015407,NC_015431
MYC_PUT	Mycoplasma putrefaciens Mput9231	NC_021083
ODO_SPL	Odoribacter splanchnicus DSM 20712	NC_015160
OSC_VAL	Oscillibacter valericigenes	NC_016046,NC_016048
PAL_PRO	Paludibacter propionicigenes WB4	NC_014734
PAR_DIS	Parabacteroides distasonis ATCC 8503	NC_009615
PED_HEP	Pedobacter heparinus DSM 2366	NC_013061
PED_SAL	Pedobacter saltans DSM 12145	NC_015177
POR_GIN	Porphyromonas gingivalis ATCC 33277	NC_010729
PRE_DEN	Prevotella denticola F0289	NC_015311
PRE_INT	Prevotella intermedia 17 NC 017860	NC_017861
PRE_MEL	Prevotella melaninogenica ATCC 25845	NC_014370,NC_014371
RAL_EUT	Ralstonia eutropha JMP134 NC 007336	NC_007337,NC_007347,NC_007348
RAL_SOL	Ralstonia solanacearum	NC_001399,NC_014308,NC_014309,NC_017558,NC_017559,NC_017589
RHO_RUB	Rhodospirillum rubrum F11	NC_017584
RIV_SPP	Rivularia PCC 7116	NC_019678,NC_019679,NC_019686
ROS_INT	Roseburia intestinalis XB6B4	NC_021012
RUM_ALB	Ruminococcus albus 7	NC_014824,NC_014825,NC_014826,NC_014827,NC_014833
RUM_BRO	Ruminococcus bromii	NC_021013
RUM_CHA	Ruminococcus champanellensis 18P13	NC_021039
RUM_OBE	Ruminococcus obeum	NC_021022
RUM_SPS	Ruminococcus	NC_021014
RUM_TOR	Ruminococcus torques	NC_021015

SEL_RUM	Selenomonas ruminantium lactilytica TAM6421	NC_017068,NC_017069,NC_017070,NC_017071,NC_017072,NC_017073,NC_017074,NC_017076,NC_017077,NC_017078
SEL_SPU	Selenomonas sputigena ATCC 35185	NC_015437
SPH_SPX	Sphingobacterium 21	NC_015277
SPI_DIM	Spiroplasma diminutum CUAS 1	NC_021833
SPI_SYR	Spiroplasma syrphidicola EA 1	NC_ 021284
SPI_TAI	Spiroplasma taiwanense CT 1	NC_021832,NC_021846
STR_PAR	Streptococcus parasangnis FW213	NC_017905
STR_PSE	Streptococcus pseudopneumoniae IS7493	NC_015875,NC_015876
STR_THE	Streptococcus thermophilus ND03	NC_017563
TAN_FOR	Tannerella forsythia ATCC 43037	NC_016610
TET_HAL	Tetragenococcus halophilus	NC_016052
TOL_AUE	Tolumonas auensis DSM 9187	NC_012691
VEI PAR	Veillonella parvula DSM 2008	NC_013520
YER_ENT	Yersinia enterocolitica palearctica 105 5R r	NC_015224,NC_015475
YER_PSE	Yersinia pseudotuberculosis IP 32953	NC_006153,NC_006154,NC_006155

Figure 1

Distribution of contig length in *strain* dataset

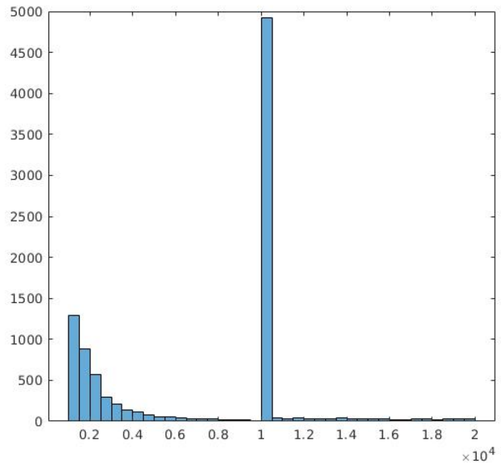

Distribution of contig length in *species* dataset

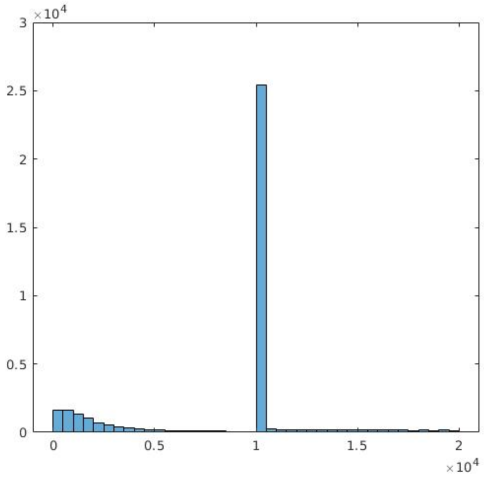

Figure 2

Assessment of precision and recall of MetaCon while varying the threshold to filter short and long contigs (dataset *strain*).

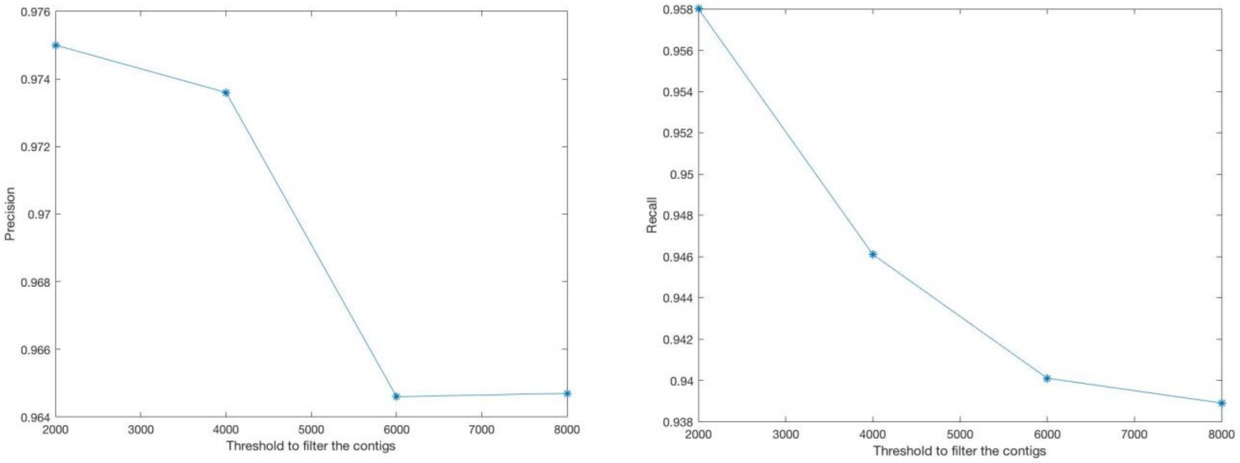

Supplement: Supplementary file 1 — Supplementary Material. (PDF 205 kb) [file 12859_2019_2904_MOESM1_ESM.pdf]
